# Oncology (2nd edn)

**DOI:** 10.1038/sj.bjc.6603485

**Published:** 2007-01-09

**Authors:** S Direkze

**Affiliations:** 1 Royal Free and University College Medical School, London, UK

The field of Oncology contributes to many medical and surgical specialities, and is also a highly specialised field in its own right. Despite appearing extensively throughout the preclinical and clinical medical course, oncology can be a daunting subject for medical undergraduates. Teaching of the subject can be spread throughout their training, and the student has to draw together these various components of the subject. Text books written on the subject are largely aimed at post-graduate level, making challenging reading for people approaching the field for the first time.

*Oncology (second edition)* by Max Watson, Ann Barrett, Roy Spence, and Chris Twelves is a text book written with the aim of providing concise and updated knowledge of adult medical and clinical oncology for undergraduate medical students, junior doctors, general practitioners, as well as hospital staff working in the care and treatment of patients with cancer.

The nineteen chapters of this text book cover an extensive array of subjects within oncology, drawing on the authors' expertise in the fields of palliative medicine, surgery, as well as clinical and medical oncology. The general structure of the text consists of a bullet-point format, highlighted text boxes emphasising ‘key facts’, ‘future possibilities’ and a summary at the end of every chapter. References are also given for further reading. Additionally, ‘stop and think’ and ‘test yourself’ sections within the chapters have been aimed at challenging students' knowledge and posing ethical and clinical decisions that occur in oncology, although not all of these sections I found were particularly useful.

The initial chapters of this book are devoted to cancer epidemiology and pathogenesis, as well as a general introduction to the clinical aspects of oncology, which include chapters on diagnosis and treatment. A particularly useful inclusion in these chapters is the explanation of various national cancer screening programmes in the UK. The chapter on cancer pathogenesis, however, is too brief and should have, perhaps, included more information on cancer biology, such as explanations of oncogenes and tumour-suppressor genes.

Separate chapters have been dedicated to chemotherapy and radiotherapy, which explain very well the mechanisms behind these therapies, their clinical indications and side effects. The chapter on chemotherapy and biological therapies includes useful tables summarising clinical information on the chemotherapy drugs, as well as explanations of the newer cancer therapies, such as the small-molecule inhibitory drugs and the monoclonal antibody therapies. The brief chapter on oncological surgery is a good addition as surgical interventions in diagnosis and treatment of cancer patients both in the curative and palliative setting is not always explained in oncology text books. A chapter on communicating with cancer patients has also been included, which gives important points and strategies to clinicians related to breaking the bad news to patients.

The remaining chapters of the book are dedicated to various cancers with in-depth chapters dedicated to the common cancers: lung, colon and breast. More general chapters cover the gynaecological, upper gastrointestinal and genitourinary cancers. Also included are chapters on tumours of the central nervous system, skin, and head and neck, as well as sarcomas. Haematological malignancies have also been well explained, in a devoted chapter. These chapters gives a good background knowledge to each of the tumours, in addition to explaining its natural history, patient signs and symptoms, diagnosis and current treatment modalities. Simplified-staging systems for the cancers have also been included and are given in a table format. The management of clinical problems related to specific cancers, such as paraneoplastic effects, are addressed, and a small chapter at the end of the text book also covers emergencies general oncological. For many of the described cancers, a typical case history of a patient has also been included to set the information given about the cancer in a clinical context.

These chapters devoted to the individual cancers follow the same concise format as seen in the introductory chapters to the text book and provide enough information to the readers that this text book is aimed at. Generally, the chapters cover many of the cancers that medical students are expected to have knowledge of. However, the subject of cancers occurring in immunosuppressed patients has not been discussed and would have been a useful additional chapter. I also felt that the chapters sometimes lack illustrations, especially of clinical signs. The Online Resource Centre that accompanies this book offers case studies and multiple-choice questions on lung, colorectal, breast, ovarian and prostate cancers, however these additional resources do not add much to the information in the text book.

Overall, I thought that this was a well-planned and written text book that successfully covers a broad range of cancers and relevant topics in oncology in concise, but sufficient detail, for its intended readers and would be a very useful guide to the clinical aspects of Oncology.

